# Remote Recruitment Strategy and Structured E-Parenting Support (STEPS) App: Feasibility and Usability Study

**DOI:** 10.2196/47035

**Published:** 2023-09-11

**Authors:** Katarzyna Kostyrka-Allchorne, Petrina Chu, Claire Ballard, Nancy Lean, Blandine French, Ellen Hedstrom, Sarah Byford, Samuele Cortese, David Daley, Johnny Downs, Cristine Glazebrook, Kimberley Goldsmith, Charlotte L Hall, Hanna Kovshoff, Jana Kreppner, Kapil Sayal, James Shearer, Emily Simonoff, Margaret Thompson, Edmund J S Sonuga-Barke

**Affiliations:** 1 Department of Child & Adolescent Psychiatry Institute of Psychiatry, Psychology & Neuroscience King's College London London United Kingdom; 2 Department of Biostatistics and Health Informatics Institute of Psychiatry, Psychology & Neuroscience King's College London London United Kingdom; 3 Academic Unit of Mental Health & Clinical Neurosciences School of Medicine University of Nottingham Nottingham United Kingdom; 4 Centre for ADHD and Neurodevelopmental Disorders Across the Lifespan Institute of Mental Health University of Nottingham Nottingham United Kingdom; 5 School of Psychology Faculty of Environmental and Life Sciences University of Southampton Southampton United Kingdom; 6 Department of Health Service and Population Research Institute of Psychiatry, Psychology & Neuroscience King’s College London London United Kingdom; 7 Solent NHS Trust Southampton United Kingdom; 8 New York University Child Study Center, Hassenfeld Children's Hospital at NYU Langone New York, NY United States; 9 Nottingham Trent University Psychology School of Social Science Nottingham Trent University Nottingham United Kingdom; 10 Department of Child & Adolescent Psychiatry Aarhus University Aarhus Denmark

**Keywords:** parenting intervention, mobile app, attention-deficit/hyperactivity disorder, ADHD, behavior problems, mobile health, mHealth, children, usability, mobile phone

## Abstract

**Background:**

The Structured E-Parenting Support (STEPS) app provides support for parents of children with elevated hyperactivity, impulsivity, inattention, and conduct problems who are awaiting clinical assessment. STEPS will be evaluated in a randomized controlled trial (RCT) within the Online Parent Training for the Initial Management of ADHD Referrals (OPTIMA) research program in the United Kingdom. Phase 1 of the OPTIMA tested the feasibility of participants’ recruitment and the app’s usability.

**Objective:**

This study aimed to adapt a digital routine clinical monitoring system, myHealthE, for research purposes to facilitate waitlist recruitment; test using remote methods to screen and identify participants quickly and systematically; pilot the acceptability of the recruitment and assessment protocol; and explore the usability of STEPS.

**Methods:**

myHealthE was adapted to screen patients’ data. Parents’ and clinicians’ feedback on myHealthE was collected, and information governance reviews were conducted in clinical services planning to host the RCT. Potential participants for the observational feasibility study were identified from new referrals using myHealthE and non-myHealthE methods. Descriptive statistics were used to summarize the demographic and outcome variables. We estimated whether the recruitment rate would meet the planned RCT sample size requirement (n=352). In addition to the feasibility study participants, another group of parents was recruited to assess the STEPS usability. They completed the adapted System Usability Scale and responded to open-ended questions about the app, which were coded using the Enlight quality construct template.

**Results:**

Overall, 124 potential participants were identified as eligible: 121 (97.6%) via myHealthE and 3 (2.4%) via non-myHealthE methods. In total, 107 parents were contacted, and 48 (44.9%) consented and were asked if, hypothetically, they would be willing to participate in the OPTIMA RCT. Of the 28 feasibility study participants who provided demographic data, 21 (75%) identified as White. Their children had an average age of 8.4 (SD 1.7) years and 65% (31/48) were male. During the primary recruitment period (June to July 2021) when 45 participants had consented, 38 (84%) participants agreed hypothetically to take part in the RCT (rate of 19/mo, 95% CI 13.5-26.1), meeting the stop-go criterion of 18 participants per month to proceed with the RCT. All parents were satisfied or very satisfied with the study procedures. Parents (n=12) recruited to assess STEPS’ usability described it as easy to navigate and use and as having an attractive combination of colors and visual design. They described the content as useful, pitched at the right level, and sensitively presented. Suggested improvements included adding captions to videos or making the recorded reflections editable.

**Conclusions:**

Remote recruitment and study procedures for testing a parenting intervention app are feasible and acceptable for parents. The parents felt that STEPS was a useful and easy-to-use digital parenting support tool.

**International Registered Report Identifier (IRRID):**

RR2-10.1186/s40814-021-00959-0

## Introduction

### Background

Attention-deficit/hyperactivity disorder (ADHD) is a neurodevelopmental condition with an estimated prevalence of between 2% and 7% of children worldwide [1,2]. It is manifested by symptoms of inattention, impulsivity, and hyperactivity and is associated with impairment across multiple life domains [3-6]. Over 40% of children with an ADHD diagnosis also display oppositional, disruptive, or defiant behaviors and meet the criteria for an oppositional defiant disorder (ODD) diagnosis [7,8]. Managing this combination of ADHD and ODD is a major challenge for parents [9]. For many parents, it is this combination that motivates them to seek help through a clinical referral to pediatric clinics or child and adolescent mental health services [10]. Parent training as recommended by the National Institute for Health and Care Excellence is the most common evidence-based intervention used to help parents manage their children’s disruptive and defiant behaviors [11].

### The Structured E-Parenting Support App

Parent training is traditionally delivered in person by clinically trained professionals. However, universal shortages in health care workforces combined with financial challenges facing public health services mean that parents face substantial waiting times in accessing this kind of support [12]. These considerable delays in access to parent training increase the risk of further deterioration of the parent-child relationship and the escalation of their the children’s problems. We have developed a digital mobile phone app to address this problem. Structured E-Parenting Support (STEPS) [13] was designed to help parents manage the disruptive and defiant behaviors of their children with elevated levels of hyperactivity, impulsivity, and inattention symptoms. In comparison with in-person support, STEPS is a low-cost, easy, and quick-to-access parenting support intervention, which provides evidence-based advice and support. Its design was inspired by an in-person parent training program, the New Forest Parenting Programme [14], with its content reflecting many years of research about parenting and child behavior [11,15-17]. Using audio-visual and graphic elements, STEPS aims to increase parents’ knowledge of children’s behavior problems, build children’s confidence, facilitate effective communication between parents and children, and provide parents with strategies and skills to better manage their children’s challenging behavior.

STEPS is currently being evaluated in a large-scale multicenter randomized controlled trial (RCT) as a way of delivering support to the families of children referred to clinical services who are on the waiting list for specialist assessment and treatment. The RCT represents the second phase of the Online Parent Training for the Initial Management of ADHD Referrals (OPTIMA; funder reference number RP-PG-0618-20003) program. Phase 1 of the OPTIMA program had 4 objectives to help the study team prepare for the future RCT, which was prospectively registered on November 18, 2021 (registration number ISRCTN16523503).

The first objective was to adapt and implement a digital platform, myHealthE, for the remote identification and screening of recently referred families [18]. This is an essential part of OPTIMA, as it ensures rapid and systematic screening of ADHD and ODD symptoms in children accepted by clinical services for a wide range of problems and from different referral sources. We asked the following question: how should myHealthE be adapted so that it can be implemented across a variety of clinical services to support OPTIMA recruitment? Objective 2 was to test the feasibility of a remote recruitment strategy incorporating myHealthE. Objective 3 was to test the wider feasibility of the full-scale trial [13]. To achieve objectives 2 and 3, we conducted a single-arm nonrandomized study. We asked two questions: (1) can the necessary number of eligible families be recruited using our remote identification and screening strategy within the planned time frame to meet the sample size requirements and provide sufficient power in the planned RCT? and (2) is the proposed RCT recruitment and assessment approach acceptable to participants? Objective 4 was to make the final, minor updates required to optimize the value of the STEPS app for families. To achieve this objective, we conducted a separate mixed methods usability evaluation of the STEPS app with a different group of parents of children aged 4 to 11 years. We asked the following questions: (1) what is the experience of parents using the STEPS app? and (2) are there ways in which they think it can be improved?

## Methods

### Adaptation of myHealthE for OPTIMA (Objective 1)

Adaptation of the platform was done based on anecdotal feedback from parents, clinicians, service managers, and research governance teams from the participating organizations. This feedback was collected through (1) group meetings with the professionals and the myHealthE team to review the plans and resources required to support implementation and (2) individual interviews with parents who are members of the OPTIMA Patient and Public Involvement and Engagement panel. The initial plan was to integrate myHealthE into local digital platforms. However, after extensive consultation with these stakeholders, it was decided that myHealthE would work better if it was a stand-alone web application. Depending on the organization’s preference, the flow of patients’ personal and clinical information between myHealthE and clinical records would occur either via manual data entry or a process of robotic process automation. Through a set of programmed instructions, the robotic process automation process allows a software robot to mimic human front-end tasks, such as manual referral data entry into myHealthE, with high efficiency [18]. This change also enhanced functionality for myHealthE users (ie, clinicians and clinical administrators) by allowing the use of a report button to generate caregiver and teacher response outcome reports, whenever needed. This new report can also be manually uploaded to the patient’s electronic clinical notes. Further, the central myHealthE team can provide group clinical outcome data as an extract on a periodic basis to support business intelligence work, such as outcome submission to the Mental Health Services Data Set, a repository of information collected via different clinical systems as part of routine patient care, and for local commissioners. Each organization received the National Health Service Digital Technology Assessment Criteria pack for myHealthE, which included data privacy impact assessment, and signed the information processing agreements with the lead organization. The success of myHealthE implementation as a gateway to STEPS access was measured in terms of the number of services that adopted the platform and five additional key performance indicators: (1) the number of parents who were onboarded onto the platform (ie, their contact details were logged, which triggered the invitation to register with myHealthE); (2) the number of those who then registered with the platform; (3) the number of those who completed the routine Strengths and Difficulties Questionnaire (SDQ) and (4) provided consent for research contact; and, finally, (5) the number of children whose parents provided consent for research contact were flagged up as *OPTIMA eligible* based on their age, referral date, and SDQ subscale scores for hyperactivity and conduct problems.

### Ethical Considerations

The observational feasibility study received ethics approval from London–Riverside Research Ethics Committee on November 17, 2020 (reference 20/LO/1173). There was no financial incentive for taking part. The STEPS app usability assessment study was approved by King’s College London PNM Research Ethics Panel (reference LRS-20/21-21359). Each participant provided written consent on the web and was given a £30 (US $38.1) shopping voucher to thank them for their time.

### Observational Feasibility Study (Objectives 2 and 3)

#### Design and Setting

This was a single-arm observational feasibility study conducted remotely [13]. Clinical recruitment sites were in England, in urban areas with catchment populations from a wide range of ethnic and socioeconomic backgrounds. The overall recruitment period lasted for 2.5 months from mid-May to the end of July 2021, with the primary recruitment period restricted to June and July 2021. The participants completed the study questionnaires and accessed the STEPS app using their private devices in their preferred setting.

#### Participants

The participants of this study were parents and teachers. Parents were recruited from 4 recruitment sites. Of these sites, 3 adopted myHealthE to facilitate trial recruitment. The fourth site used nondigital methods (not myHealthE) to obtain consent for research contact and screen for ADHD- and ODD-type symptoms. One further site agreed to support the pilot and feasibility study but did not recruit any participants. Inclusion criteria specified that participants were parents of new referrals (on waitlist no longer than 6 calendar months; the initial definition for “new referrals” referring to children being on the waitlist for less than 3 months was modified during the study, and this modification was approved on June 25, 2021) aged 5 to 11 years who passed the initial triage and had been accepted onto the assessment waiting list but had not yet received a diagnosis of ADHD. The parent had to have rated their child as having a high level of ADHD symptoms (a score≥8) and conduct problems (a score≥4) during routine clinical screening with the SDQ, a brief questionnaire used to measure symptoms of psychopathology in children and adolescents [19]. Following an initial conversation with researchers, parents were excluded if they lacked access to a suitable electronic device, had an insufficient level of English language, or if their child was under local authority care. Parents who met the eligibility criteria were invited to participate in the study. Parents who agreed to participate provided written web-based consent, including, in most cases, consent for the team to contact their child’s general practitioner and school. Reasons for not enrolling in the study were recorded by the study team.

There were no inclusion or exclusion criteria for teachers, but researchers were required to obtain parents’ permission for contacting teachers.

#### Testing the Feasibility of Remote Recruitment

The feasibility of recruiting a sufficient number of participants for the RCT was assessed by asking each study participant a *feasibility question*. More specifically, participants who consented to take part in the observational feasibility study were read a script explaining the proposed design and procedures of the phase 2 OPTIMA RCT and how it would differ from the current feasibility study. It was explained to participants that taking part in the phase 2 RCT would involve a longer time commitment than the current feasibility study and that they would be randomly assigned to either a group that received the STEPS app straight away or a group that remained on the waitlist without access to the app. Following this explanation, participants were asked to respond “yes” or “no” to whether they would be willing to participate in such a study *in principle*.

Power calculations for the planned RCT in phase 2 of the OPTIMA program indicated that 13 participants per month would need to be recruited to the trial over the 27-month recruitment period (n=352) for the trial to have sufficient power to test for hypothesized differences in the primary outcome. A more conservative stop-go requirement of recruiting 18 participants per month was adopted in the observational feasibility study to consider the potential differences between agreeing in principle in the current feasibility study and actually consenting to take part in the OPTIMA RCT. The rate of participants agreeing per month was calculated as the number of participants agreeing in principle to take part in the RCT during the primary feasibility study recruitment period (June to July 2021) with the associated 95% Poisson CI (using an immediate CI command in Stata [version 17; StataCorp] specifying a Poisson distribution). We also calculated the proportion of participants who agreed by dividing the number of participants who agreed by the number of participants who were recruited and then multiplying the resultant value by 100.

#### Piloting the Acceptability of the Recruitment and Assessment Protocol

The acceptability of the recruitment and assessment protocol was evaluated by asking parents to provide ratings of satisfaction with the consenting procedures and web-based data collection via the exit questionnaire. In addition, we measured the following: (1) the time taken to complete the remote consenting procedures; (2) the proportion of participants who completed all outcome questionnaires within 7 days of receiving a link to the web-based questionnaires out of the number of participants who were in the study; (3) the number of reminder emails about completing the outcome questionnaires sent to parents by the research team; (4) the proportion of participants who completed the adverse event questionnaire within 7 days of receiving a link to the web-based questionnaire out of the number of participants who were in the study; and (5) the mean number of reminder emails about completing the adverse events questionnaire sent to parents by the research team. We also assessed the feasibility of collecting data from children’s teachers by measuring the time needed to identify teachers and the proportion of teachers who returned the outcome questionnaire within 7 days of receiving a link to the web-based questionnaire out of the number of teachers who were recruited.

The piloted measures included parent-completed questionnaires, specifically prebaseline measures to characterize the sample and outcome measures, and a teacher-completed questionnaire. The prebaseline measures included the Eyberg Child Behavior Inventory [20]; Social Communication Questionnaire [21]; and ADHD subscale of the Swanson, Nolan, and Pelham Rating Scale [22]. The outcome measures included the O’Leary Parenting Scale [23,24]; the ODD subscale of the Swanson, Nolan, and Pelham Rating Scale [22]; the Parental Sense of Competence Scale [25]; the Caregiver Strain Questionnaire [26]; and a demographic questionnaire, which asked questions about the parent’s gender, educational level, employment status, income, ethnicity, and relationship status and the number and ages of other children in the household and whether they had received an ADHD diagnosis. Finally, parents were also asked whether they had received parent training of any type or had any mental health difficulties that required clinical treatment in the previous 6 months. The teachers completed only the ODD subscale of the Swanson, Nolan, and Pelham Rating Scale [22].

#### Procedure

Information on the child’s age, sex, ADHD symptoms, and conduct problems was derived from the existing referral information. Parents completed questionnaires on the web using Qualtrics (Qualtrics International Inc), a secure web-based data collection platform. Each participant was enrolled in the study for approximately 4 weeks. Those who completed the baseline questionnaires were then emailed instructions on how to access the STEPS app. Importantly, they were informed that the use of the app was optional and were not prompted to use it (the plan for the definitive trial was to monitor and prompt use). Two weeks after the baseline questionnaires were completed, parents were sent a link asking them to complete an adverse events questionnaire on the web. Four weeks after the baseline questionnaires were completed, parents were debriefed and asked to complete an exit questionnaire assessing satisfaction with the remote consenting process and the web-based outcome and adverse events data collection procedures. Parents who consented to have their child’s teacher contacted provided the teacher’s contact information so that a teacher information sheet and consent statement could be sent to the teacher, along with a link to the web-based teacher questionnaire.

#### Data

We recorded baseline demographic information, scale scores from the prebaseline and baseline outcome questionnaires, the time taken to complete the study procedures, the number of reminder emails sent, satisfaction survey responses, and the number of participants who completed all web-based questionnaires within 7 days (as a proportion of the number of participants who were given access). The number of people who took up the invitation to download the app was also recorded. Safety data were summarized as the number of adverse events and the number of people who experienced adverse events. The prebaseline and baseline questionnaire score summaries are presented in Multimedia Appendix 1.

### STEPS App Usability Assessment (Objective 4)

#### Participants

Participants were 12 parents (all female) of children aged 4 to 11 years recruited from the general population through advertisements on social media, as well as through the OPTIMA Patient and Public Involvement and Engagement panel. One further parent took part in the initial session but did not complete the entire study and was, therefore, subsequently excluded from the sample.

#### Measures

The measures used to fulfill objective 4 were an adapted System Usability Scale [27] and open-ended questions asked in a think-aloud session and follow-up semistructured interviews. The System Usability Scale is a 10-item questionnaire that uses a mix of positively and negatively worded items designed to assess the usability of a digital tool (eg, the ease of use, a user’s confidence in using the tool, and the perceived amount of technical support that would be required to use the tool). Responses were made on a 5-point scale ranging from 1 (“strongly disagree”) to 5 (“strongly agree”). To calculate the overall usability score, 1 is subtracted from the score of each positively worded item and the score of each negatively worded item is subtracted from 5 to give a score ranging from 0 to 4 for each item, where higher scores reflect more positive responses. These item scores are then summed and multiplied by 2.5 to give a total score ranging from 0 to 100.

Open-ended questions probed the participants’ first impressions about the app, including its look, feel, and navigation, as well as elicited more detailed views on the overall experience of using the app and on each of the elements contained within the app.

#### The STEPS App

**Figure 1:**
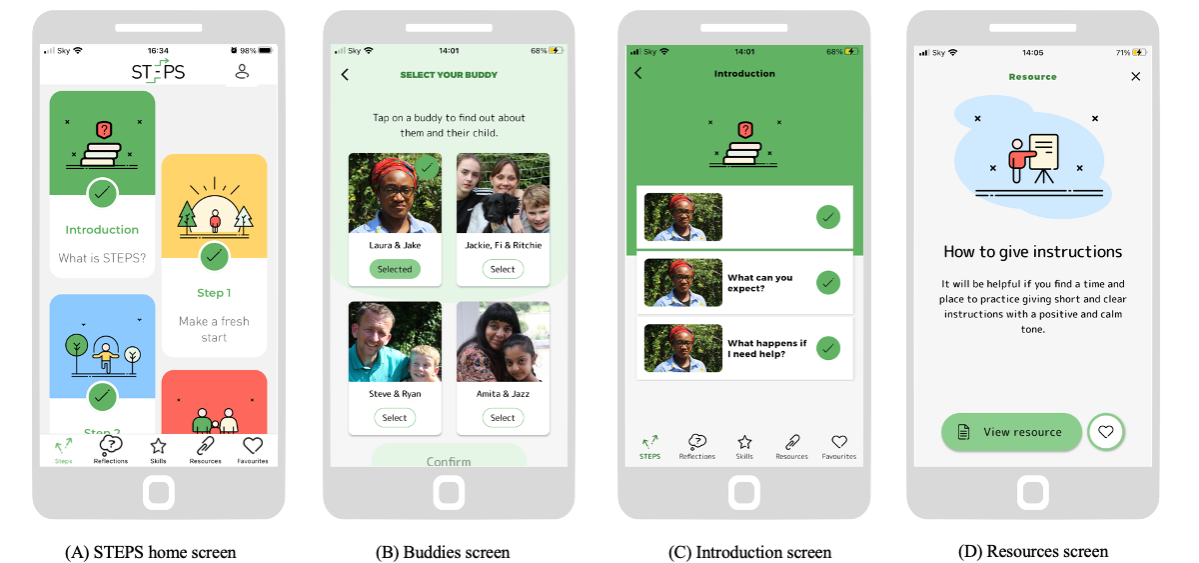
Examples of the Structured E-Parenting Support (STEPS) app screens: (A) Home screen. (B) Buddy selection screen. (C) Introduction screen. (D) Resources screen.

A detailed description of the STEPS app is provided in Multimedia Appendix 2. Because of the unguided nature of the STEPS app, several design features were implemented in it to improve engagement. First, a “Buddy,” a parent played by an actor, accompanies the user on their journey through STEPS. Upon registering with the app, each user is directed to a screen that provides brief video vignettes of the 4 available Buddies (Figure 1) and is asked to select 1. Buddies can be changed an unlimited number of times during subsequent use of the app. Within each module, the selected Buddy provides an overview of the content and then recaps the key points covered. Second, a brief introductory module needs to be completed, where the selected Buddy provides a brief overview of the content and gives advice on how to use STEPS (eg, take a break for a few days between the modules and record reflections). Third, the app has a linear structure to allow users to build up their parenting skills, with a clear visual distinction between the completed modules (and components within each module; Figure 1) and those that are yet to be completed. Users can also make a note of the content that they particularly like or would like to revisit for quick access by including it among their favorites. Finally, the content is delivered in short, accessible, and “bite-size” pieces, that is, individual videos or audio clips are not longer than 3 minutes to keep users engaged and avoid overwhelming them with too much information.

#### Procedure

Each participant took part in 2 remote video sessions facilitated by a trained researcher. In the first, think-aloud, session, participants were asked to download the app, complete a few simple navigation tasks (eg, select and change a Buddy, watch a video, record a reflection, and save an item to favorites), and speak out loud what came to their mind as they completed these tasks. They also answered questions regarding their first impressions of STEPS. The second, follow-up, session was scheduled approximately a week later, and the participants were instructed to use the app as much as they could during the intervening period. In the second session, participants were first asked questions about their general mobile phone use and then asked more detailed questions about their views on the STEPS app. All sessions were audio and video recorded. The automatically generated transcripts were checked, and any identifying information was removed. The participants were also emailed a link asking them to complete the System Usability Scale on the web via Qualtrics.

#### Data Analyses

The total System Usability Scale and individual item scores were summarized using means and SDs. Qualitative data from the think-aloud and follow-up sessions were analyzed using the template analysis method [28]. This is a style of thematic analysis that requires the development of a structured coding template. To align with the themes discussed in previous relevant studies, we adopted a prespecified coding template. Specifically, responses were coded using the Enlight quality construct template developed by Baumel et al [29]. This template was derived from a systematic review of quality rating criteria for digital health interventions, tested with both mobile phone–based and web-based interventions, and includes the following core constructs:

1. Usability: the ease of learning how to use the app and the ease of using it properly
2. Visual design: the look and feel of the app
3. User engagement: the extent to which the app’s design attracts users to use it
4. Content: the content provided or learned while using the app
5. Therapeutic persuasiveness: the extent to which the app is designed to encourage positive behavior changes
6. Therapeutic alliance: the ability of the app to create an alliance with the user to motivate change
7. Potential: a subjective evaluation of the app’s potential to benefit its target users

## Results

### myHealthE Adaptation (Objective 1)

myHealthE was used by 2 child and adolescent mental health services and 1 local authority early behavioral help service. At the end of the overall feasibility recruitment period (July 31, 2021), a total of 1024 patients were onboarded onto the platform, including 952 (92.97%) new referrals and 72 (7.03%) existing patients. Of the 952 new referrals, 768 (80.7%) registered with the platform, 649 (68.2%) completed the routine SDQ, and 308 (32.4%) provided consent for research contact. Finally, 121 children whose parents provided consent for research contact were flagged up as *OPTIMA eligible*.

### Observational Feasibility Study

#### Participant Characteristics

**Figure 2:**
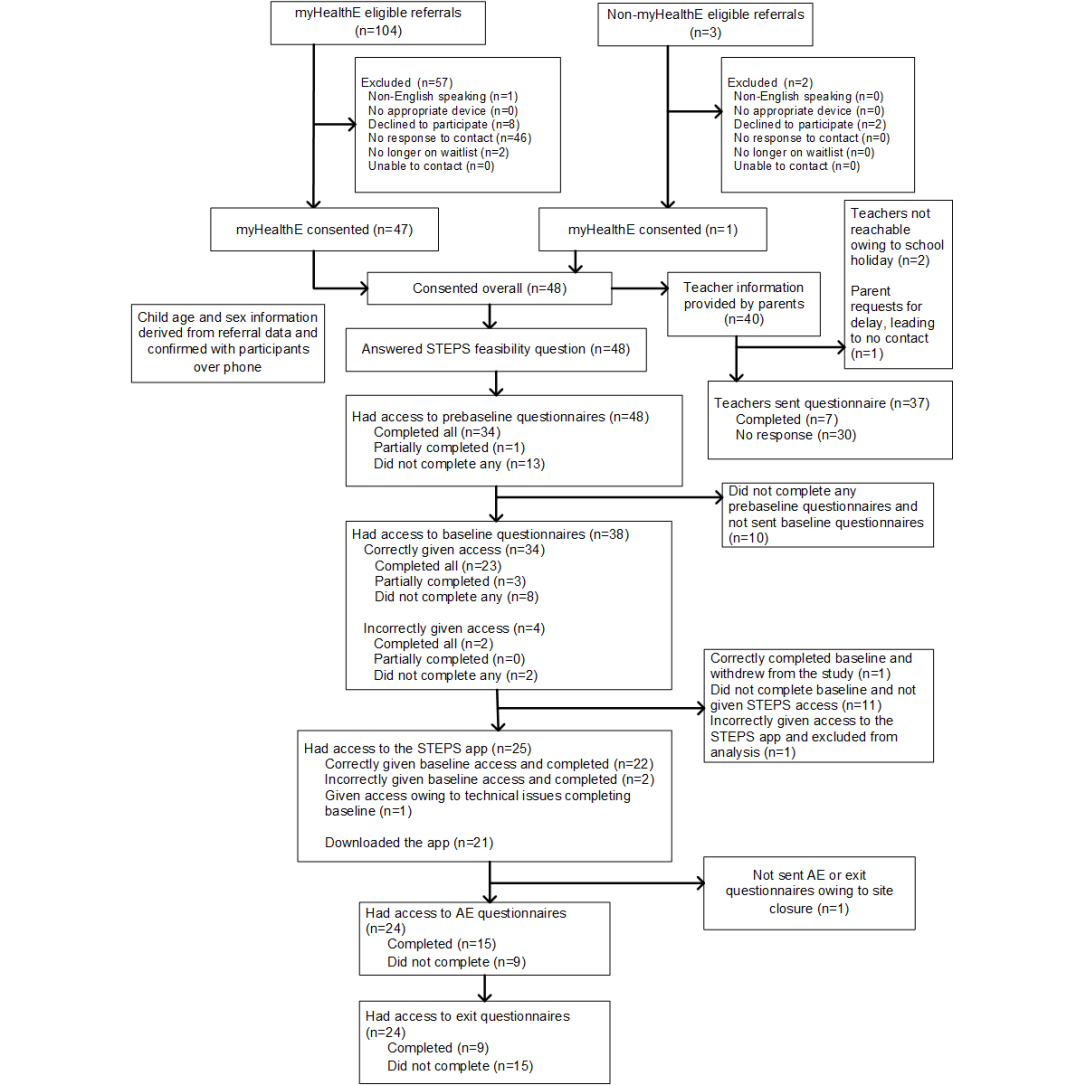
Observational feasibility study CONSORT (Consolidated Standards of Reporting Trials) flowchart. AE: adverse event; MHE: myHealthE; STEPS: Structured E-Parenting Support.

Of the 107 eligible referrals, who were approached with an invitation to participate in the study, 104 (97.2%) were identified by myHealthE, and the remaining 3 (2.8%) were identified via non-myHealthE methods. Of the 107 referrals, 48 (44.9%) consented to participate in the study (Figure 2). All 48 participants answered the feasibility question about willingness to participate in principle in an OPTIMA RCT and were given access to the prebaseline questionnaires, which were completed by 34 (71%) participants. Then, 38 participants received access to the baseline questionnaires (4 participants were incorrectly given access), which were completed by 25 (66%) participants. These 25 participants were provided with access to the STEPS app (1 was given access erroneously). Of the 25 people who were given access to the app, 21 (84%) downloaded it. Of the 24 participants provided with the adverse events questionnaire about medical and psychological events and difficulties (1 was not provided with the questionnaire owing to the recruitment site closure), there were 15 (62%) completers. Information about the adverse events reported in the study is included in Multimedia Appendix 3. The same 24 participants were provided with exit questionnaires, with 9 (38%) completers. Only 1 (2%) participant out of the total 48 formally withdrew from the feasibility study owing to a house move. Of the 48 parents, 40 (83%) provided teacher information, and 37 (92%) teachers were contacted. A total of 8% (3/40) of teachers were not contacted (2/40, 5% owing to school holidays preventing contact and 1/40, 2% owing to a parent requesting a delay to search for an email address of the teacher, which was never provided). Only 7 (19%) out of the 37 teachers completed the questionnaire within the 1-week response window.

The mean age of the children in the feasibility participant sample (n=48) was 8.4 (SD 1.7) years, and 31 (65%) out of 48 were male. The mean SDQ hyperactivity subscale score was 9.5 (SD 0.7), and the mean conduct problem subscale score was 6.2 (SD 1.7). Of the 28 parents who provided responses on the demographic questionnaire, 21 (75%) were White. Of the 28 parent respondents, 16 (57%) were married or in a long-term relationship and 16 (57%) had completed General Certificate of Secondary Education, Certificate of Secondary Education, Ordinary Level, or equivalent qualifications. All participants’ demographic information and children’s ADHD symptoms and conduct problems scores are presented in Table 1. A summary of the clinical outcome measure scores is provided in Multimedia Appendix 1.

**Table 1:** Characteristics of the observational feasibility study participants.

Characteristic				Values
**Children (n=48)**				
	**Age (years)**			
		Mean (SD)	8.4 (1.7)	
		Median (IQR)	9 (7-10)	
	**Sex, n (%)**			
		Female	17 (35)	
		Male	31 (65)	
	**SDQ^a^ hyperactivity subscale score**			
		Mean (SD)	9.5 (0.7)	
		Median (IQR)	10 (9-10)	
	**SDQ conduct problem subscale score**			
		Mean (SD)	6.2 (1.7)	
		Median (IQR)	6 (5-7)	
**Parent participants (n=28)**				
	**Ethnicity, n (%)**			
		Black or Black British	6 (21)	
		White British, Irish, or other	21 (75)	
		Mixed race White and Black or Black British	1 (4)	
	**Sex, n (%)**			
		Female	28 (100)	
	**Education, n (%)**			
		No formal qualifications	9 (32)	
		Completed GCSE^b^ or CSE^c^ or O-levels^d^, equivalent	16 (57)	
		Completed post-16 vocational course	2 (7)	
		Undergraduate or professional qualification	1 (4)	
	**SES^e^ (£^f^; annual income levels), n (%)**			
		<16,000	9 (32)	
		16,000-29,999	7 (25)	
		30,000-59,999	11 (39)	
		≥60,000	1 (4)	
	**Marital status, n (%)**			
		Single (never married)	9 (32)	
		Married or in a long-term relationship	16 (57)	
		Widowed	1 (4)	
		Divorced	1 (4)	
		Separated	1 (4)	

^a^SDQ: Strengths and Difficulties Questionnaire.

^b^GCSE: General Certificate of Secondary Education.

^c^CSE: Certificate of Secondary Education.

^d^O-levels: ordinary level.

^e^SES: socioeconomic status.

^f^£1=US $1.27.

#### Findings

##### Can We Recruit a Sufficient Number of Participants Using Our Remote Strategy to Meet the Power Needs of the OPTIMA RCT?

All 48 participants answered the feasibility question, with 41 (85%) agreeing in principle to take part in an RCT. Focusing on the primary recruitment period (June to July 2021), 38 (84%) out of 45 participants agreed in principle to take part in an RCT. This was a rate of 19 (95% CI 13.5-26.1) participants per month, which exceeded the conservative stop or go criterion of 18 participants per month set a priori. We note that the lower limit of the CI excludes 13 per month, that is, the less conservative estimate of the number needed from the power calculation. This suggests we will likely be able to recruit >13 families per month.

##### Is the Recruitment and Assessment Protocol Acceptable?

The mean time from a service accepting a referral onto a waitlist to the completion of remote consenting by the participant was 51 (SD 40) days. The mean parent rating of satisfaction with consenting procedures was 4.6 (SD 0.5) out of 5; a total of 4 (44%) out of 9 parents were “satisfied,” and 5 (56%) out of 9 were “very satisfied.”

Of the 38 parents who provided baseline outcome data, 23 (61%) completed all the questionnaires within 7 days. The mean number of reminder emails about web-based data completion sent to parents was 1.1 (SD 1.6; median 0, IQR 0-3). The mean parent rating of satisfaction with web-based data collection was 4.4 (SD 0.7) out of 5; overall, 1 (11%) out of 9 participants selected a “neutral” response, 3 (33%) out of 9 participants were “satisfied,” and 5 (56%) out of 9 participants were “very satisfied.” Finally, 9 (38%) out of 24 parents completed adverse event questionnaires within 7 days, and the mean number of reminder emails about adverse event questionnaire completion sent to parents was 0.67 (SD 0.70). The average time from the date when participants consented to the date when teachers were identified was 1.5 (SD 6.3; median 1, IQR 0-1) days, and 7 (19%) out of the 37 teachers returned questionnaires within 7 days.

### STEPS Usability Evaluation

#### Participant Characteristics

Of the 12 participants who were recruited specifically for the STEPS usability study, 8 (67%) worked part time or full time, and 4 (33%) were stay-at-home parents. All participants reported using mobile phones frequently for various purposes, such as communication, leisure, banking, navigation, or shopping. None of them reported any general difficulties with using mobile phone technology. Overall, 33% (4/12) of participants reported lesser mobile phone use on weekends than on weekdays. The main reason cited for reduced weekend mobile phone use was “family time.”

#### Findings

##### System Usability Scale Analysis

Parents rated the app’s usability as very high; the overall STEPS usability score on the System Usability Scale was 94.8 (SD 4.8) out of 100. Individual item responses also showed that participants’ experience of using STEPS was positive (Multimedia Appendix 4).

##### Template Analysis of Open-Ended Questions About the App

*Usability*: all participants found the app simple to use and straightforward to navigate. Many commented that the app was “intuitive” and that navigation was “obvious” and “self-explanatory.” Detailed quotes are presented in Multimedia Appendix 5. Some participants attributed the ease of use to the fixed linear structure of the app. However, 1 parent found the need to complete the modules in a fixed order frustrating. Participants found the clear visual distinction between completed and not-completed steps helpful in navigating the app and commented that simple language also improved the usability of the app. Participants made suggestions for improvements, for example, providing captions for videos and transcripts and making the recorded reflections editable. Some also wanted to receive more information about Buddies and their roles.

*Visual design*: the parents provided very positive feedback about the look and feel of the app. In particular, they commented on the attractive combination of colors and visual design features:

I really like the look of it, I really like the design and the graphics, they look really classy, but they also just look very professional.

Some participants used the word “friendly” to describe the look and feel of the app:

It’s simple to use and kind of feels nice and modern and friendly.

Finally, 1 participant’s comment also suggested that the structure of the app created very positive first impressions about the look and feel:

I found it made sense and it flowed well. I like the way it’s laid out. I think it’s going to be easy to use on my first impression, it’s certainly not daunting, it’s quite clear to understand.

*User engagement*: comments from several participants suggested that receiving information in short “bite-size” pieces was the key to successful engagement with the app:

...and it was also bite-sized amount of information which I liked. It wasn’t throwing loads of information at you at once, because obviously that’s just overloads yourself [sic], especially if you’re busy. I found that quite useful.

Parents also mentioned that receiving a notification from their Buddy (push notification) served as a useful reminder to log back into the app:

If I had like a really busy day and I hadn’t looked at it [the app] and then I got a notification from my buddy that said about Steps, it was like a little reminder, oh yes, I need to log on and do that and that was actually was [sic] really helpful. It’s quite motivating that you’ve got that little prompt.

The variety of formats, including videos, audio clips, and text resources, made the app more engaging:

I liked that there was [sic] different elements, it wasn’t all just videos, there was some audio. I like the versatility of it and just that there was [sic] different elements, it wasn’t just consistently the same thing.

Although the possibility of accessing the app at any time and at any place is created by general smartphone affordances rather than benefits specifically limited to STEPS, users highlighted such accessibility as an important feature:

I think it’s really useful just to kind of have it in your pocket all the time and to have it readily available.

Several participants wanted to receive more information regarding the Reflections feature of the app, specifically, in relation to the privacy and confidentiality of what is recorded there by users. One of the participants said that they would “filter” what they would record in Reflections, rather than freely express their thoughts, if these recordings would be shared with others. Suggestions were also made that although some reflections should be private, it would be useful to be able to choose to share some of the recordings, for example, with a clinician or another professional as “evidence of the child’s behaviour or reflections on what’s worked well.”

*Content*: participants commented that the content was pitched at the right level and presented in a sensitive way:

I liked the content I thought it [expert videos] was really well written in that it gave you the information that you needed, but it was in a very understandable format and I like again the fact it was a video very relatable, not patronising, I thought it was good.

The variety of examples included in the app made the content applicable to a wide range of parents, as one participant noted:

It’s quite nice when you first open it [examples] that you can just see a range of children and a range of problems of looking like a menu for things and you can sort of spot which ones.

The participants highlighted the importance of including children’s perspectives in the examples and provided further suggestions on how to give children more presence in the app. For example, this could be achieved by including real stories of children whose parents used the app successfully or by creating sections within the app that could be completed by both a parent and child.

*Therapeutic persuasiveness*: participants commented that the aims of STEPS were realistic and that the advice was straightforward to implement:

I like the fact that it starts at the very beginning and about reconnecting with your child and your relationship with your child and that it works through. I thought the aims were also realistic.

One participant provided an example of how working through the STEPS resources motivated them to reflect on their own situation and to act to effect change:

I did find them [resources] useful. Oh yeah, it was the making quality time for yourself. So, reading through that I actually bought myself a yoga mat in the week ‘cause I thought, ok I’m going to sit with my headphones on, forget everything and do that. So yeah, reading through that has made me realise if I’m not at my best because I’m always busy and I’m always doing everything.

Including children’s perspectives in the app’s examples was also noted as a factor that may help motivate change:

Examples from the children and giving their perspective on things, and I wasn’t expecting that, and actually I found that really useful and quite kind of it’s almost moving, going and I’m not doing it on purpose and generally don’t hear when my mum is telling me to turn off my gaming and that I thought was really, really kind of makes you go oh gosh, yeah [sic]. So, that I think was brilliant having those little bits in, because they are only very short aren’t they? I think really quite powerful in a way.

Finally, one of the parents suggested how reflections could be used to motivate changes by giving users space to write an action plan which advice or skills they want to implement:

The other thing I thought is, the reflections bit at the moment is just getting you to think about stuff but, I wondered from a kind of behaviour change perspective, if whether sometimes that could be used to prompt people to commit to things that they want to try, like what are you going to try this week? Which of these suggestions would be good for you? And how and when are you going to try them out? ‘Cause that would then act as something to stop it just being something that you spent 10 minutes having a quick listen to or look at and then don’t do anything with.

##### Therapeutic Alliance

*Therapeutic alliance*: several participants commented that their Buddies managed to create a sense of personal connection and relatability:

I think the buddy system is probably my favourite. I’ve never come across anything like that before...It makes it easier to connect I think with the buddies.

They also commented that the inclusion of various examples helped demonstrate that the app’s aims were achievable, and the context was relatable:

I thought they [examples] were really good because they were very accurate and they also made it easier to relate to the Steps programme, because the examples were realistic and were quite common problems that parents would be going through.

The expert videos also came across as friendly and knowledgeable, and the content was delivered using an accessible language:

That’s really good. I like that, she’s a great speaker. She’s very calm. She’s not overcomplicating. She’s not using loads of jargon and everything which I hate when you start getting into these sorts of things.

However, the comment from one of the parents highlighted an important risk that is inherent to unguided parenting interventions, that is, working through the app may create uncomfortable reflections about one’s parenting and lead to the feeling of self-blame:

I think the lady, the doctor that was describing it she was fantastic and she kept it very simple, but sometimes when I was listening to it I felt like oh, not I know that [sic], but it felt like a bit like ok, so everything that’s happening in my life...it felt like it’s my fault, like I’ve not been the best parent up to now.

*Potential*: participants noted the gap in the provision of help and support for parents and thought that the app could help address some of those unmet needs:

I think it’s a great idea. I have to say I think there is really a gap in parental support. So actually, to have something that is available absolutely all the time at any point when you need it, I think is really good. I think it’s a great way to try and support parents’ cause.

They commented that the app could be helpful to a wide range of parents, regardless of whether their child has received a diagnosis or is on the waitlist:

I know a lot of parents who’ve already had their diagnosis and have literally just been given a diagnosis and said congratulations off you go now and that’s it [sic]. With no help or support. Just there you go, and they would really benefit from this. So, it would be great if it was more widely available. But also, yes for that for that waiting period it’s horrendous and you do know nothing.

Finally, the app could also be helpful to parents of children who do not have clinical-level needs:

I think every parent is looking for something like this, because we all struggled. And of course, parenting is such a difficult thing and there is lots of scope in it, you know to improve yourself, so I think it is giving me a very positive vibe [sic]. In helping myself and my child to manage the behaviour and the steps don’t look complicated.

## Discussion

### Principal Findings

This paper reported on phase 1 of the OPTIMA research program. It had 4 objectives concerning the adaptation and testing the feasibility of the screening and waitlist recruitment strategy facilitated by the myHealthE platform, piloting the acceptability of the proposed remote recruitment and assessment protocol, and exploring the usability of the STEPS mobile app to optimize its functionality for parents. Overall, our findings were positive and demonstrated that the planned recruitment strategy and assessment protocol were feasible and acceptable to participants. Usability data also supported the use of STEPS to provide support for families on the child health services waitlist and provided useful recommendations for minor modifications to the app.

Our findings showed that myHealthE can be successfully adapted and used across the 3 different child health services in the United Kingdom. To support timely implementation, the original plan to make the platform interoperable with the local clinical patient records systems had to be modified, and myHealthE was implemented as a stand-alone desktop application that could be accessed via a web browser. This adaptation did not compromise the platform’s clinical utility in terms of monitoring patient-reported outcomes, as individual reports could be easily generated by the clinic staff. Crucially, myHealthE provided a systematic and efficient way for researchers to screen and identify eligible families from the waitlist of the participating services without the need to involve members of the clinical care team. Traditional approaches require that a patient’s (or, in the case of patients aged <16 years, their parent’s) consent for research contact be obtained by a clinician and recorded in clinical notes. These notes are subsequently manually screened to identify potentially eligible participants. Such a process not only is time consuming, resulting in delays in contact, but also means that clinicians act as the main gatekeepers to providing access to research opportunities. For families on the waitlist who have very limited or no contact with clinicians until their first assessment appointment, this could be a substantial barrier to being involved in research. Compared with these traditional approaches, myHealthE permitted a straightforward and convenient way of obtaining consent for research contact and facilitated the timely and efficient recruitment of participants from the service waitlist into the study.

Our remote recruitment strategy was also successful. During the primary feasibility study recruitment period, 45 participants consented to take part in the study, and 38 (84%) of them agreed *in principle* to take part in the RCT, exceeding our conservative assumption of 18 participants per month. This suggests that we should comfortably achieve our planned RCT recruitment target of 13 participants per month. This finding is important for 2 reasons. First, meeting trial recruitment targets is essential to ensuring the success of a trial. Second, recruitment from health services can be very challenging (even more so when participants are recruited from the waitlist), and a substantial proportion of trials either experience delays leading to higher research costs or are stopped owing to poor recruitment [30]. It could be that using remote approaches, such as the one adopted in this study, which give participants the maximum flexibility of completing consenting procedures at the time and place that are convenient to them, helps overcome some of the key barriers to successful recruitment. Importantly, remote recruitment and assessments were also acceptable to parents. Those who provided exit questionnaire data were either satisfied or very satisfied with the study procedures. In addition, the feasibility study provided an important learning opportunity for the research team. We uncovered some errors in the study procedures, such as participants receiving access to web-based questionnaires or the app when they should not have. Becoming aware of these potential issues during the feasibility study will help us to develop clear operating procedures to minimize the risk of making errors in the RCT.

Finally, the STEPS app received high usability ratings, and parents provided very positive feedback about the app. Participants found the app easy to navigate (mainly owing to its clear linear structure) and visually attractive. They appreciated the easy-to-understand language used in the app, which was clear of psychological jargon, and found it useful to have information presented in varied formats (ie, text, video, and audio). Many parents emphasized that it was helpful to have information presented in short, “bite-size” pieces that could be accessed when they had a few spare minutes (eg, when waiting to collect their child after school). Although some parents found the functionality that allowed them to record reflections useful, a few expressed concerns about the confidentiality of recording their private thoughts within the app. The key recommendations for enhancing the app included making improvements to the process of app registration, making resources shareable, improving video playback, and adding captions to videos.

The use of digitally mediated approaches to identification, recruitment, and data collection is efficient from the researchers’ point of view and convenient for many participants. We established that myHealthE provided an effective method for screening and identifying participants and that our remote recruitment and assessment strategy was feasible and acceptable. However, adopting digital methods may have resulted in a sample that overrepresented individuals with a high level of digital skills. Moreover, access to myHealthE and the STEPS app relies on having access to a device that is connected to the internet, which some families may not have. Ultimately, these families would not be able to access and potentially benefit from the intervention. Research suggests that it is often those already at a disadvantage because of education and employment opportunities, income, disability, or geographic location who are most likely to be excluded from digital access [31]. If not managed carefully, this may further widen existing health inequalities. Furthermore, we should acknowledge that the eligibility requirement that study participants have a reasonable understanding of English has inevitably led to the exclusion of parents from linguistically (and culturally) diverse backgrounds. Researchers adopting digital recruitment methods and those developing mobile phone interventions should consider the impact of digital competence and language exclusion on the generalizability and reach of their findings.

### Conclusions

This study demonstrated that digital screening and remote recruitment from child clinical services’ waiting lists are feasible. They are also timely and efficient and minimize the burden on clinical teams, which are typically substantially involved when nondigital recruitment methods are used. Such procedures are also acceptable to participants. Usability data indicate that STEPS has the potential to deliver parenting support to parents of children with ADHD-type symptoms.

## Appendix

Multimedia Appendix 1 Prebaseline and baseline questionnaire score summaries.

Multimedia Appendix 2 The Structured E-Parenting Support app.

Multimedia Appendix 3 Adverse event and serious adverse event summary metrics.

Multimedia Appendix 4 The System Usability Scale individual item scores.

Multimedia Appendix 5 Additional quotes from the usability interviews.

## Abbreviations

ADHD: attention-deficit/hyperactivity disorder
ODD: oppositional defiant disorder
OPTIMA: Online Parent Training for the Initial Management of ADHD Referrals
RCT: randomized controlled trial
SDQ: Strengths and Difficulties Questionnaire
STEPS: Structured E-Parenting Support
