# Supplementary material for: Remote Recruitment Strategy and Structured E-Parenting Support (STEPS) App: Feasibility and Usability Study
Source: JMIR Pediatr Parent. 2023 Sep 11;6:e47035. doi: 10.2196/47035 (PMC10520770; doi:10.2196/47035)
Supplement: Multimedia Appendix 1 [file pediatrics_v6i1e47035_app1.docx]

## Multimedia Appendix 1: Pre-baseline and baseline questionnaire scores summaries.

## Supplemental Table S1. Pre-baseline and baseline questionnaire scores summaries.

| **Pre-baseline clinical measures** | **Missing (% of 48)** | **Overall** |
| --- | --- | --- |
| ECBI Intensity total - median (IQR) | 13 (27.1%) | 137.0 (124.0-147.0) |
| ECBI Intensity total - mean (SD) | 13 (27.1%) | 133.3 (18.6) |
| SCQ total - mean (SD) | 13 (27.1%) | 19.4 (7.0) |
| ADHD subscale (SNAP-IV) - mean (SD) | 13 (27.1%) | 2.4 (0.5) |
| **Baseline clinical measures** | **Missing (% of 38)** | **Overall** |
| ODD subscale (SNAP-IV) - mean (SD) | 10 (26.3%) | 2.1 (0.6) |
| Laxness subscale (The Parenting Scale) - mean (SD) | 11 (28.9%) | 3.3 (1.1) |
| Over-reactivity subscale (The Parenting Scale) - mean (SD) | 11 (28.9%) | 3.1 (1.2) |
| Hostility subscale (The Parenting Scale) - mean (SD) | 11 (28.9%) | 1.9 (1.1) |
| PSOC total - mean (SD) | 11 (28.9%) | 62.7 (8.7) |
| CSQ Global - mean (SD) | 10 (26.3%) | 9.8 (2.2) |
