# Supplementary material for: Remote Recruitment Strategy and Structured E-Parenting Support (STEPS) App: Feasibility and Usability Study
Source: JMIR Pediatr Parent. 2023 Sep 11;6:e47035. doi: 10.2196/47035 (PMC10520770; doi:10.2196/47035)

Structured E-Parenting Support (STEPS)

What is STEPS?

STEPS is a parenting support intervention delivered in the form of a digital application (app).

What is STEPS aiming to do?

STEPS has been designed to decrease conflict in the family by reducing levels of children's oppositionality and defiance. It helps parents to be more effective and self-confident in managing these problems. It is designed to be particularly helpful for the parents of children who are temperamentally more difficult to manage, such as those with attentional and impulse control problems. It is aimed at supporting parents of primary school-aged children.

How was STEPS developed?

STEPS is evidence based. Inspired by the New Forest Parenting Programme, a face-to-face parent training intervention, its content has been shaped by the latest research about parenting and child behaviour as well as many years of clinical experience.

How is STEPS implemented?

STEPS can be accessed through a variety of devices. Parents can move through the content (steps) at their own pace and at any time of day. The order of the steps is fixed (see 'How is STEPS structured?'), although there is a degree of choice within each step. STEPS is an unguided intervention, which means there is no personal clinical support for parents progressing through STEPS. The content is delivered mainly using short videos and audio clips. During onboarding each parent will register with the app and choose one of four 'buddies' – a parent character played by an actor, who will accompany them on their STEPS journey.

How is STEPS structured?

STEPS has two preparatory modules, "onboarding" and "introduction", followed by eight separate intervention modules (steps). These steps must be followed in order.

What are the eight steps?

1. **Make a fresh start –** Encourages parents to see their child and themselves in a new, more positive way.
2. **Look after yourself** – Emphasizes how important it is for parents to find time for themselves and to make links with other parents.
3. **Get co-operation** – Explains ways parents can communicate more effectively with their children.
4. **Build confidence** – Highlights how important it is for parents to create situations in which they can praise their child.
5. **Keep it cool** – Helps parents to think of ways they can avoid losing their temper with their children when they are being difficult.
6. **Guide & support** – Shows how parents can help their children navigate around difficult situations where they may find themselves getting upset.

7. **Structure & boundaries you can trust** – Demonstrates how vital it is that everyone signs up to and follows the house rules.
8. **Reduce conflict and improve behaviour** – Explains how using rewards and sanctions can promote better behaviour in children.

What elements are included in the steps?

Each step has a similar structure and includes the following common elements:

i) **Aims**: sets out the themes to be covered in a step presented by Buddy.
ii) **Science**: provides a short and easy to understand video-description of the evidence behind a step's message presented by a real-life expert.
iii) **Examples:** videos of parents (played by actors) discussing their parenting experiences.
iv) **Skills:** audio presentations of specific skills in simple short sentences accompanied by graphical illustrations.
v) **Reflections:** a chance for parents to write or talk about their experiences.
vi) **Resources:** downloadable aides and guides relating to each step.

How long will STEPS take to complete?

This will depend on the pace and frequency of usage. However, each of the 8 steps are designed to take about 20 minutes if completed in one go.

How will engagement be encouraged?

The app has an attractive design and is easy to use. The use of buddies and pre-recorded prompts automatically sent to parents' devices will help maintain engagement and remind users to reconnect.

What happens if parents are having difficulty?

There will be support for parents encountering technical difficulties. For any serious clinical concerns, parents will be provided with contact details for the clinical service to whom they were referred as well as direct contact details for crisis services.

What information will be recorded?

Each parent will be assigned a unique ID, which will be used to link app usage data with the offline study information. Usage data including the number of steps, elements completed, the amount of time parents were engaged with the app and the time of day the app was accessed will be collected.

Who created STEPS?

STEPS concepts and content was designed by Edmund Sonuga-Barke, David Daley, Johnny Downs, Hanna Kovshoff, Jana Kreppner and Margaret Thompson with Samuele Cortese and Cathy Laver-Bradbury providing advice. STEPS visual design and digital implementation was completed by TOAD with funding provided to Sonuga-Barke by the South London & Maudsley NHS Trust. Videos were produced by Eye Witness Productions Ltd. funded by Solent NHS Trust. Special thanks go to Catherine Thompson for her work on an earlier prototype – *New Forest On-Line*.

Journey through STEPS

Home screen

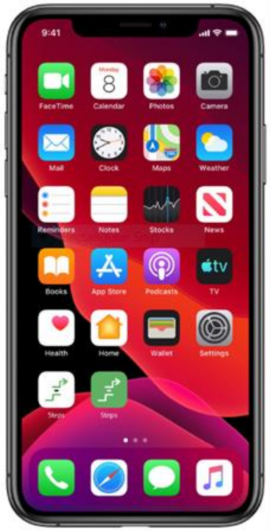

Welcome

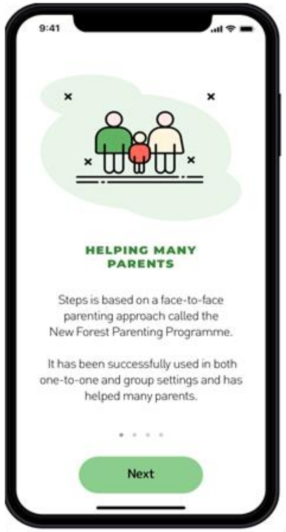

Onboarding

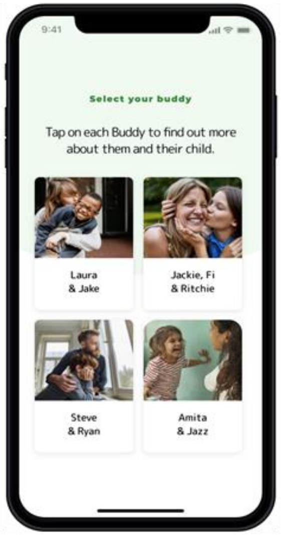

Introduction

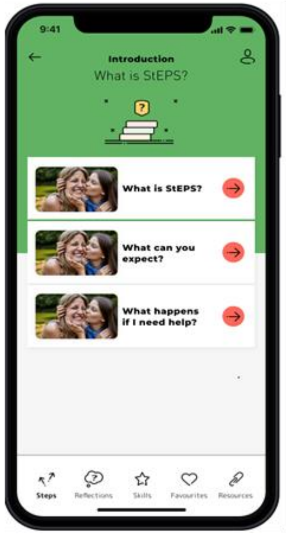

Module menu

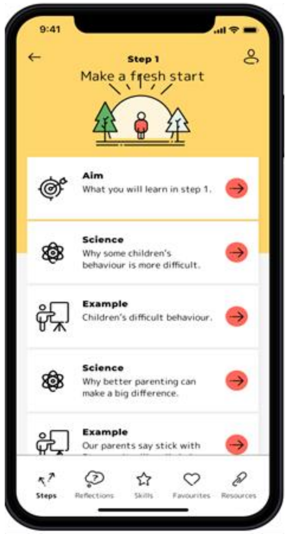

Aims

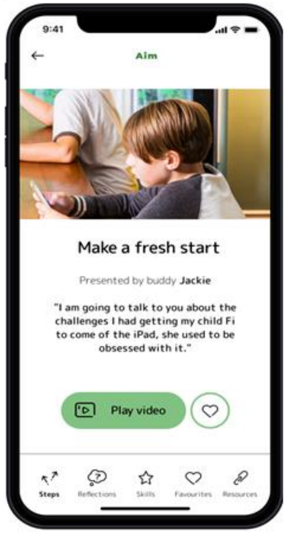

Science

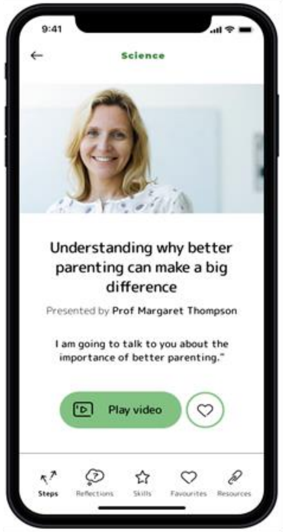

Reflection

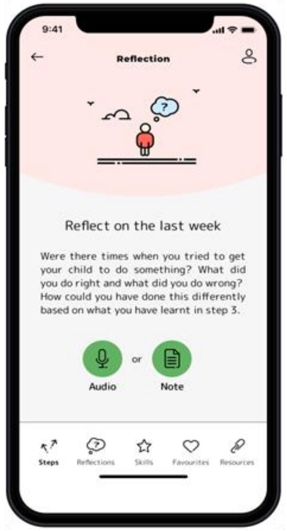

Skill

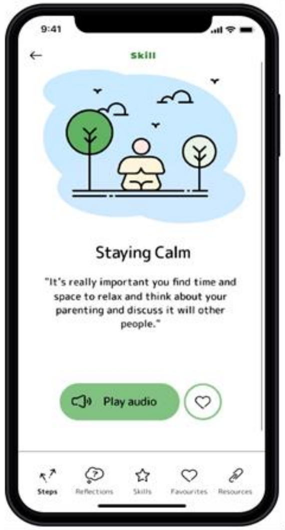

Completed step

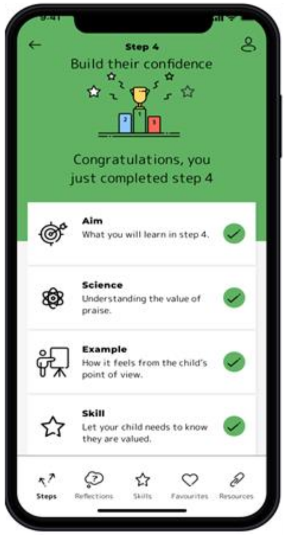

Supplement: Multimedia Appendix 2 [file pediatrics_v6i1e47035_app2.pdf]
