# Supplementary material for: Remote Recruitment Strategy and Structured E-Parenting Support (STEPS) App: Feasibility and Usability Study
Source: JMIR Pediatr Parent. 2023 Sep 11;6:e47035. doi: 10.2196/47035 (PMC10520770; doi:10.2196/47035)
Supplement: Multimedia Appendix 3 [file pediatrics_v6i1e47035_app3.docx]

**Multimedia Appendix 3. Adverse event (AE) and serious adverse event (SAE) summary metrics.**

A summary of adverse and serious adverse events is in Supplemental Table S2. There were no serious unexpected, related events suspected to be related to app use, and none of the adverse events reported led to participant withdrawal. There were six reports of serious adverse events. All six serious adverse events were categorised as not related to the intervention. One participant reported four different serious adverse events (one for their child and three for themselves), and the other two serious adverse events reports were made by two different participants (one for their child and the other for themselves). We note that due to an oversight, the adverse events form only allowed the collection of one adverse event record each for the parent and child though some parents made use of the available free text box to provide information about more than one event.

## Table S1. Adverse event (AE) and serious adverse event (SAE) summary metrics.

| **Number of events (participants reporting)** | **Overall number of events (number of people)§** | | **Number of events by individual affected (number of people)§** | | | | |
| --- | --- | --- | --- | --- | --- | --- | --- |
| **Overall Category** | **AEs (n = 16)** | **SAEs**  **(n = 6)** | **Child AEs (n = 7)** | **Child SAEs**  **(n = 2)** | **Parent AEs**  **(n = 9)** | | **Parent SAEs**  **(n = 4)** |
| Child refusal to go to or exclusion from school | 2 | 0 | 2 | 0 | 0 | 0 | |
| Significant deterioration in child/sibling/parent behaviour or wellbeing | 10 (8) | 0 | 4 (4)* | 0 | 6 (6)* | 0 | |
| Significant medical issue in child/parent/other immediate family member | 4 (3) | 6 (3) | 1 | 2** | 3 (2) | 4 (2**) | |
| **Event subcategory:** |  |  |  |  |  |  | |
| Endocrine | 0 | 1 | 0 | 1 | 0 | 0 | |
| Musculo-skeletal | 1 | 2 | 0 | 0 | 1 | 2 | |
| Neurological | 0 | 1 | 0 | 0 | 0 | 1 | |
| Psychological | 12 (10) | 1 | 6 (6)* | 0 | 6 (6)* | 1 | |
| Dermatological | 1 | 0 | 1 | 0 | 0 | 0 | |
| Other – Injury | 0 | 1 | 0 | 1 | 0 | 0 | |
| Other - Lack of energy | 1 | 0 | 0 | 0 | 1 | 0 | |
| Other - Vitamin D deficiency | 1 | 0 | 0 | 0 | 1 | 0 | |

**§**If a single number is presented without parentheses, the number of events and the number of people are the same for this cell. If a number is presented with parentheses, more than 1 event was reported for that category/subcategory by a participant. The number presented in parentheses is the number of individual participants reporting a particular event. In this sample, there were 10 reports of significant deterioration in child/sibling/parent behaviour or wellbeing made by 8 different participants. We have recently become aware that the form used to gather adverse events was set up in error to only allow one event to be entered per person per category (the six categories were physical child/participant, mental/behavioural child/participant and family relationships/daily activities child/participant), so the above may not be an accurate count of events.
