# Supplementary material for: Remote Recruitment Strategy and Structured E-Parenting Support (STEPS) App: Feasibility and Usability Study
Source: JMIR Pediatr Parent. 2023 Sep 11;6:e47035. doi: 10.2196/47035 (PMC10520770; doi:10.2196/47035)
Supplement: Multimedia Appendix 4 [file pediatrics_v6i1e47035_app4.docx]

### **Multimedia Appendix 4. The System Usability Scale individual item scores.**

Table S1. Means, standard deviations (SD) for the System Usability Scale, n = 12. 1= strongly disagree; 5 = strongly agree.

| **SUS item** | **Mean (SD)** | **Median (min-max)** |
| --- | --- | --- |
| I think that I would like to use this app frequently. | 4.3 (0.9) | 4 (2-5) |
| I thought the app was easy to use. | 4.8 (0.4) | 5 (4-5) |
| I found the various functions in this app were well integrated. | 4.6 (0.5) | 5 (4-5) |
| I would imagine that most people would learn to use this app very quickly | 4.8 (0.5) | 5 (4-5) |
| I felt very confident using the app. | 5.0 (0.0) | 5 (5-5) |
| I found the app unnecessarily complex. | 1.2 (0.4) | 1 (1-2) |
| I think that I would need the support of a technical person to be able to use this app | 1.0 (0.0) | 1 (1-1) |
| I thought there was too much inconsistency in this app. | 1.3 (0.5) | 1 (1-2) |
| I found the app very cumbersome to use. | 1.1 (0.3) | 1 (1-2) |
| I needed to learn a lot of things before I could get going with this app. | 1.0 (0.0) | 1 (1-1) |
