# Supplementary material for: Remote Recruitment Strategy and Structured E-Parenting Support (STEPS) App: Feasibility and Usability Study
Source: JMIR Pediatr Parent. 2023 Sep 11;6:e47035. doi: 10.2196/47035 (PMC10520770; doi:10.2196/47035)
Supplement: Multimedia Appendix 5 [file pediatrics_v6i1e47035_app5.docx]

## Multimedia Appendix 5. Additional quotes from the usability interviews.

## Table S1. Additional quotes from the usability interviews.

| Construct | **Quotes** |
| --- | --- |
| Usability | **Linear structure - positive**  “I like the fact that you're actually forced to finish one before you can do the next, so I'd probably be darting all over the place and you get the full experience”  “The navigation was very easy and you in fact don't have to do anything, it's very much spoon feeding. I felt like after completing one thing you directly move to another step and move to another part of that same section”.  **Linear structure – negative**  “I wanted to skip some steps and go directly to Step 5, but I couldn't so that was a bit irritating for me.”  **Clear visuals**  “I like the way things become colour, the colour changes to make it clear where you are and which things you're not on. It stops you going to things that you're not supposed to go to, but it also makes it quite clear, quite intuitive.”  “It's really easy, because of the way it's laid out, the colours, the way you go from one to the other”.  **Language:**  “…not too much complicated jargon which can really bog you down.”  “Yeah, navigation I think it's all there and it is self-explanatory, and I like the words that its uses. There isn't so many psychological words.”  “Very logical […] and it is in plain English.” |
| Visual design | “I like all the pictures, I like all the colours, it’s very easy, it doesn't feel heavy”.  “It's nice. It's colourful […] The look and feel of it is quite nice.”  “It's very professional but friendly […] it seems really sort of calming and gentle.” |
| User engagement | **Bite-sized pieces of information**  “So, I think for parents who are rushing around everywhere and don't have much time because you're doing everything in really short chunks, that's really good. You can literally go oh, I've got a couple of minutes I can put that one on and then later and I'll do the other one. Just the fact that everything is broken down into small bite sized chunks is really good.”  “I liked that each section was broken down so that you could come do it as it's done in almost chapters, step by step.”  **Notifications**  “I was surprised pleasantly when I got the notification, oh, I have to do this, you know? So that was a good thing, getting the notification.”  **Variety of formats**  “I really like that it's that there are videos are not loads of text.”  **Accessibility at any time:**  “The fact that I was just able to just do it at any time really was quite useful.”  “Like while I'm on the train going to school, I can use even there, in that 20 minutes time I could browse through it.”  **Privacy and confidentiality:**  “So, I would prefer a little bit more guidance about what reflection is for, and also presumably that the researchers won't download it and analyse it, just making clear that it’s just a private tool to help you remember things or link it to your own experiences or something like that.” |
| Content | **General - positive**  “I would say all the content was meaningful without sort of being over the top or too simplistic or it sort of felt like it was gauged appropriately.”  “I think this app is just yeah, it's fantastic for really gently introducing parents into a different way of parenting, but without criticising it's great.”  **General – negative**  “I think within the videos, there was a lot of information to grasp in one video. I love that the videos were short like four or five minutes, but then I think it was too much within a single video, especially when the professor was speaking, it was too much in just two minutes, so I was kind of lost and I couldn't grasp everything or memorize everything.”  **Examples – children’s perspective**  “I liked in some of them it's not just the parents’ giving examples, it's the kids giving examples. And some of them were honest and real it seemed in terms of I don't like it when mums do this, so they do this and you know, it's pretty confronting to hear it from a child. So it wasn't just parents perspective, so I really like that.” |
| Therapeutic alliance | **Buddies:**  “I felt like the lady in the video, Jackie, is very engaging, very warming and it's not a doctor, it's not a scientist talking to you, it's someone else probably in my boat or whatever who I can relate to.”  “I think that's quite a good idea because I guess it [buddies] maybe gives you a bit more of a personal connection with them, it's not just some sort of not faceless, but you know if you've had some input int who you think you might like, I guess it probably gives you a bit more of a connection with what they're saying.”  **Examples:**  “I liked about that some of them [examples] I could relate to. I think you got the title of it, so it kind of gives you an idea of where I could go to if there was something particularly happening in my life situation.”  **Expert videos:**  “The very first one [expert video] and it just felt really nice. Really nice opener, cause it was really validating and kind of optimistic and blame free, cause it was explaining the nature nurture thing quite well.” |
